# Dietary Patterns Influence Chronic Disease Risk and Health Outcomes in Older Adults: A Narrative Review

**DOI:** 10.3390/nu17243910

**Published:** 2025-12-13

**Authors:** Jordan A. Gunning, Madeline F. Converse, Behzad Gudarzi, Wanees Lotfallah, Susan B. Racette

**Affiliations:** College of Health Solutions, Arizona State University, Phoenix, AZ 85004, USA; jagunning@asu.edu (J.A.G.); bgudarzi@asu.edu (B.G.);

**Keywords:** dietary patterns, chronic disease, nutrition, lifestyle, aging, ultra-processed foods, health outcomes, frailty, metabolic health, cognitive function

## Abstract

The global population is aging rapidly and the prevalence of age-related noncommunicable diseases is increasing. Favorable dietary patterns have the power to reduce the risk or progression of various age-related chronic diseases, including obesity, hypertension, cardiovascular disease, type 2 diabetes, several types of cancer, and some neurodegenerative diseases. In contrast, adverse dietary patterns may contribute to the onset or progression of many chronic diseases or their risk factors. A diet rich in wholesome, nutrient-dense, minimally processed foods, such as a Mediterranean-style diet, may promote health and prevent disease through its abundance of antioxidants, fiber, omega-3 fatty acids, and micronutrients. Conversely, a diet high in nutrient-poor and ultra-processed foods may accelerate disease onset and progression by promoting inflammation and affecting metabolic pathways adversely. This narrative review summarizes the literature from clinical trials and large population-based studies on protective dietary patterns and adverse dietary patterns that influence risk of cardiovascular disease and related risk factors, cancer, Alzheimer’s disease and related dementias, type 2 diabetes, frailty, and liver disease.

## 1. Introduction

**Figure 1:**
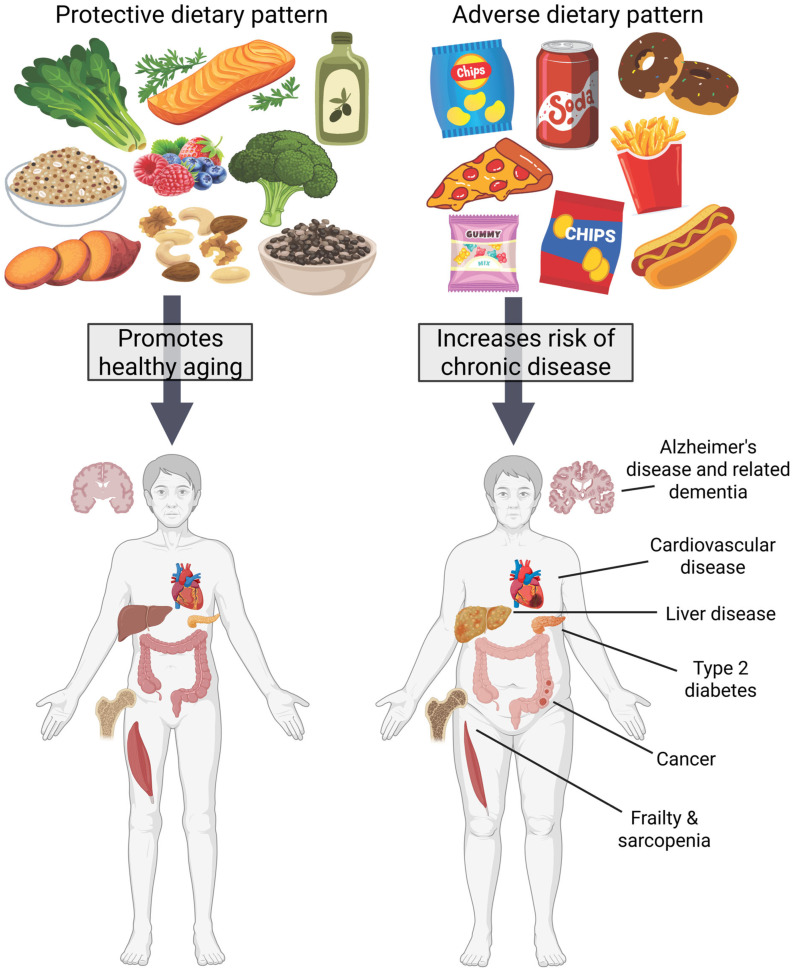
Dietary patterns contribute to healthy aging or disease development. A diet rich in whole, unprocessed foods such as vegetables, fruits, olive oil, legumes, nuts and seeds, and oily fish can contribute to the maintenance of normal cognition, metabolic health, and muscle mass and strength. A diet high in ultra-processed foods such as packaged snacks, processed meats, and sugar-sweetened beverages may contribute to systemic inflammation, poor metabolic health, neurodegeneration, cancer, frailty and sarcopenia. Created in Canva and BioRender. Gunning, J. (2026) Accessed 7 November 2025. https://BioRender.com/i2qwuqo.

The global population is aging. Consequently, the prevalence of age-related noncommunicable diseases, including cardiovascular disease (CVD), Alzheimer’s disease (AD), and cancer, is increasing [1,2]. More concerningly, perhaps, adults are becoming increasingly unhealthy and burdened by chronic illness at younger ages, presumably driven by unfavorable lifestyle patterns [3]. These trends place a growing and significant burden on healthcare systems, patients, caregivers, and families. There is mounting evidence that lifestyle habits can either contribute to or delay the onset and progression of noncommunicable disease. A healthful diet is among the most important lifestyle habits to prevent or delay the onset of chronic disease (Figure 1).

Three high-quality dietary patterns that prioritize unprocessed, health-promoting foods are the Mediterranean diet, the Dietary Approaches to Stop Hypertension (DASH) diet, and the Mediterranean–DASH Intervention for Neurodegenerative Delay (MIND) diet [4,5]. These dietary patterns emphasize consumption of vegetables, fruits, lean proteins, nuts and seeds, and legumes, and are therefore rich in fiber, omega-3 fatty acids, polyphenols, and micronutrients [6].

There are several indices that quantify diet quality. The Mediterranean Diet Score (MDS) is an index that quantifies how well a person’s diet aligns with a Mediterranean diet [7]. The Healthy Eating Index (HEI)-2015 is a quantitative measure that assesses how well a diet aligns with the Dietary Guidelines for Americans [8]. In brief, the Dietary Guidelines for Americans emphasize intake of vegetables, fruits, lean protein, grains, dairy, and oils, while limiting intake of added sugars, saturated fats, and sodium [9]. A higher HEI-2015 score indicates a diet that more closely aligns with the Dietary Guidelines for Americans [8]. Notably, the Alternative Healthy Eating Index (AHEI) is an alternative to the HEI; key differences are the inclusion of alcohol and micronutrients, as well as a greater emphasis on healthy fats [10]. The dietary inflammatory index (DII) is a quantitative measure of diet-associated inflammation and is widely used in nutrition research; a lower DII score indicates a diet with relatively few inflammatory foods and more anti-inflammatory foods and is therefore favorable [11]. Importantly, many studies utilize the energy-adjusted-DII (E-DII), which adjusts the DII score based on total calorie intake.

Overconsumption of ultra-processed foods (UPFs) is common in the United States and other developed countries. Shockingly, over half of all calories consumed in the United States come from UPFs [12]. The NOVA classification system categorizes food into one of four groups: unprocessed or minimally processed (e.g., vegetables, grains, beans), processed culinary ingredients (e.g., oils, salt, honey), processed foods (e.g., cheese, fresh bread, canned foods), and ultra-processed foods (e.g., commercially produced snacks, ready-to-eat meals, candy) [13]. UPFs generally are high in calories, added sugars, saturated and oxidized fats, sodium, additives, and/or preservatives. Moreover, UPFs are designed to be appealing and easy to overconsume and therefore contribute to obesity and a host of other health problems [14,15]. The Western diet is characterized by high consumption of UPFs, including red and processed meats, sugar-sweetened beverages, and packaged snacks, and is low in vegetables, fruits, and whole grains [16]. Diets high in UPFs tend to be low in nutritional quality, dietary fiber, and micronutrients [17].

Chronic inflammation is associated with numerous diseases and often contributes to pathology [18]. Consumption of UPFs and specific dietary components often found in UPFs is a major contributor to systemic inflammation [19]. Low-grade chronic inflammation is a hallmark of aging and is often referred to as “inflammaging” [20]. Lifestyle habits may accelerate or decelerate the onset of inflammaging. An inflammatory diet is defined as one high in processed meat, refined sugars, deep-fried foods, red meat, and ultra-processed foods. Conversely, an anti-inflammatory diet is high in polyphenols, isoflavonoids, fiber, micronutrients, and omega-3 fatty acids, and thus consists mostly of vegetables, fruits, nuts, seeds, legumes, seafood, and herbs and spices.

This narrative review summarizes the literature on protective and adverse dietary patterns that may influence the development of specific age-related chronic diseases, including cardiovascular disease (CVD), cancer, Alzheimer’s disease (AD), type 2 diabetes (T2D), frailty, and liver disease (Table 1). The importance of these conditions is highlighted by their high prevalence and adverse impact on disability, quality of life, and mortality. Statistics indicate that CVD, cancer, AD, and T2D are ranked as the 1st, 2nd, 6th, and 7th leading causes of death, respectively, in the United States [21]. This review presents data from clinical trials and large population-based studies; while the focus was older adults, we included studies in which the average participant age was at least 45 y. Preclinical and animal studies were excluded.

## 2. Cardiovascular Disease

CVD remains the leading cause of death worldwide [83]. CVD and CVD-related risk factors are highly influenced by lifestyle patterns. The American Heart Association’s Life’s Essential 8™ Cardiovascular Health Score encompasses key factors that influence cardiovascular health in two main categories: health behaviors (diet, physical activity, nicotine use, sleep health) and health factors (body mass index [BMI], blood lipids, blood glucose, and blood pressure) [84].

### 2.1. Protective Dietary Patterns

An anti-inflammatory diet may be protective against cardiovascular morbidity and mortality, and mounting evidence indicates that a higher DII score is associated with CVD risk. The MedLey study, a randomized controlled trial (RCT) assessing changes in DII scores among 152 Australian older adults, randomized participants to consume either a Mediterranean diet or their habitual diet for six months. The Mediterranean diet group had a reduction in DII scores at two and four months, whereas no change was observed in the habitual diet group [22]. Moreover, E-DII scores at baseline were positively associated with BMI, abdominal adiposity, systolic blood pressure, and waist-to-hip ratio, and inversely associated with high-density lipoprotein (HDL) cholesterol [22].

An analysis of 92,383 participants in the Nurses’ Health Study and the Health Professionals Follow-up Study revealed that individuals who consumed more than 7 g/d of olive oil, a major component of the Mediterranean diet, had a 19% lower risk of CVD mortality than those who never or rarely consumed olive oil during a 28 y follow-up period [25]. Interestingly, they also found that replacing 10 g/d of margarine, butter, mayonnaise, or dairy fat with 10 g/d of olive oil was associated with up to 16% lower risk of CVD mortality [25]. The CORDIOPREV study was an RCT assessing the cardioprotective effects of a low-fat diet versus a Mediterranean diet in 1002 participants with coronary heart disease [23]. In a fully adjusted model, the risk of major cardiovascular events was 28% lower in the Mediterranean diet group compared to the low-fat diet group [23]. An multicenter intervention trial of 7447 adults aged 55–80 y at high risk for CVD compared a Mediterranean diet supplemented with nuts, a Mediterranean diet supplemented with extra-virgin olive oil, and a control diet [26]. After a median follow-up of 4.8 y, participants in the Mediterranean diet with nuts or Mediterranean diet with olive oil groups had a 28% and 31% lower incidence of major cardiovascular events (HR = 0.72 and 0.69), respectively, compared to participants in the control diet group [26]. Additionally, a secondary analysis of dietary patterns in the ASPirin in Reducing Events in the Elderly (ASPREE) randomized trial and the ASPREE Longitudinal Study of Older Persons (ALSOP) cohort revealed that the highest quartile of MDS was associated with less obesity (i.e., fewer participants with BMIs > 33 kg/m^2^), less central adiposity, and less hypertension compared to the lowest quintile [24].

A cross-sectional analysis of 128,612 participants aged ≥60 y in the UK Biobank revealed an inverse association between vegetable protein and C-reactive protein (CRP) and between fiber intake and CRP [27]. A longitudinal analysis of 19,913 participants with frailty in the UK Biobank who were followed for a median of 11.2 y found that consumption of unprocessed poultry was protective against cardiovascular mortality, as well as cancer mortality and all-cause mortality [28].

The gut microbiome has essential roles in metabolism, energy control, and maintaining a healthy weight [85]. Diet patterns control the composition, function, and diversity of the gut microbiome, and modulation of the gut microbiome may be a predominant mechanism by which a healthy diet reduces disease risk. Specifically, diets that emphasize plant-based foods, fermented foods, and fiber, increase diversity and promote production of short chain fatty acids, thereby reducing inflammation [86]. A study using NHANES data from 41,193 participants aged ≥20 y reported an inverse relationship between scores on the dietary index for gut microbiota and CVD risk [87]. Further, for every one-unit increase in this dietary index for gut microbiota score, CVD risk decreased by 3%, suggesting that higher dietary adherence elicits greater benefits [87].

Food modeling studies highlight the predicted health benefits of replacing specific foods with more healthful options. The European Prospective Investigation into Cancer and Nutrition (EPIC)-Norfolk Study modeled replacement of 2.5% of energy from SFAs from different meat products with SFAs from dairy products. They reported that replacing SFAs from total meat with SFAs from dairy was associated with 11% lower CVD incidence during a median 21 y follow-up period among 21,841 participants [29]. Additionally, replacing SFAs in processed meat with cheese was associated with 23% lower incidence of CVD, 23% lower incidence of coronary artery disease, and 19% lower incidence of stroke [29]. Further, replacing SFAs in red meat with cheese was associated with 14% lower incidence of CVD [29].

### 2.2. Adverse Dietary Patterns

Western diet patterns increase CVD risk. Processed meat is a major source of diet-induced inflammation. An analysis of 19,913 participants with frailty aged 40–69 y from the UK Biobank revealed that consuming processed meat more than 4.0 times per week was associated with a 25% higher risk of cardiovascular mortality when compared to consuming processed meat 0–0.9 times per week during the 11.2 y follow-up period [28]. A cross-sectional analysis of UK Biobank data from 128,612 participants aged ≥60 y showed a positive association between animal protein and CRP and total dietary protein and CRP [27]. Interestingly, high animal protein intake combined with low fiber intake resulted in an average 0.65 mg/L higher CRP concentration when compared with low animal protein and high fiber intake [27]. In the Brazilian Longitudinal Study of Adult Health (ELSA-Brasil), a prospective cohort study of more than 13,000 adults aged 35–74 y followed for nearly 8 y, a one standard deviation (SD) higher intake of total UPF was associated with elevated gains in weight and waist circumference (defined as ≥90th percentile) and higher incidence of metabolic syndrome and hypertension [30]. In addition, a one SD higher consumption of processed meats and distilled alcoholic beverages was associated with elevated gains in weight and waist circumference, while a one SD higher consumption of sweetened beverages was associated with a higher incidence of metabolic syndrome and hypertension over an 8 y follow-up period [30].

An analysis of 90,631 participants from the UK Biobank found that those in the highest tertile of UPF consumption had a 13% higher risk of CVD and a 14% higher risk of cardiometabolic multimorbidity compared with participants in the lowest tertile over an 11.6 y follow-up period [31]. Additionally, every 10% increment in UPF intake was associated with a 3% higher CVD risk and a 7% higher risk for cardiometabolic multimorbidity. Genetic predisposition also influences the magnitude of the impact of UPF intake on health outcomes. UK Biobank participants in the highest tertile of UPF intake and highest tertile of polygenic risk score risk had a 118% higher incidence of CVD and 79% higher incidence of hypertension compared to participants in the lowest tertile for UPF intake and lowest tertile of polygenic risk score [31].

The Whitehall II cohort study followed 7138 older adults for 16–19 y and identified participants as having either low, moderate, or high UPF intake; the highest UPF consumers had a 23% higher risk of CVD and a 32% higher risk of coronary heart disease during the 16 y follow-up compared to low UPF consumers [32]. Likewise, a secondary analysis of the ASPREE and ALSOP studies reported that among 12,416 participants, the prevalence of dyslipidemia was significantly higher in participants in the highest quartile of UPF consumption when compared to those in the lowest quartile [24]. Additionally, a cross-sectional study in 490 older patients with T2D found significantly higher total cholesterol and significantly lower HDL cholesterol with every 20 g increase in UPF consumption, illustrating one potential mechanism by which UPF intake increases CVD risk [33].

Chronic inflammation is a significant risk factor for CVD. A study exploring the impact of UPF consumption on systemic inflammation in 1986 adults aged 46–70 y reported that participants in the highest quartile of UPF intake had significantly higher C-reactive protein, interleukin-6, tumor necrosis factor-alpha, leptin, resistin, white blood cells, neutrophils, and neutrophil-to-lymphocyte ratio, and significantly lower adiponectin than those in the lowest quartile [34]. In a prospective cohort study of 3013 Chinese adults aged ≥65 y without CVD followed for a median of 16.8 y, participants in the highest tertile of DII score had a 43% higher risk of CVD incidence and a 45% higher risk of CVD mortality compared to those in the lowest tertile [88]. Further, participants in the highest tertile of DII score also had significantly higher serum homocysteine, delineating a potential mechanism by which higher DII score may increase CVD risk [88]. An analysis of National Health and Nutrition Examination Survey (NHANES) data from participants with metabolic syndrome aged ≥40 y supported this relationship between dietary inflammatory factors and CVD; each unit increase in DII score was associated with a 15.7% higher risk of cardiovascular mortality, and participants in the highest DII tertile had an 81% higher risk of cardiovascular mortality compared to those in the lowest tertile [89]. A different analysis of 16,512 NHANES participants aged ≥60 y found similar results, with participants in the highest DII quartile having 28% higher odds of CVD and 19% higher odds of hypertension than those in the lowest quartile [90].

## 3. Cancer

Despite substantial advancements in early detection and treatment options, cancer remains the second leading cause of death in the United States and a leading cause of death worldwide [91]. It is estimated that 50% of cancers are preventable, and up to 10% of cancer cases may be attributable to poor dietary habits, including low calcium intake, low fiber intake, and low fruit and vegetable intake [92]. Dietary strategies have the potential to reduce cancer risk, improve response to traditional cancer treatments, and alleviate side effects of cancer treatments that impact quality of life.

### 3.1. Protective Dietary Patterns

Nutrition habits have consistently been linked to gastrointestinal cancers, particularly colorectal cancer (CRC). Of note, a high-quality diet fosters growth of beneficial gut bacteria that can produce short chain fatty acids, which can reduce inflammation and improve gut barrier integrity [86], highlighting a potential mechanism by which diet may impact CRC risk. A study of 92,383 participants in the Nurses’ Health Study and Health Professionals Follow-up Study reported that participants consuming > 7 g of olive oil daily had a 17% lower risk of cancer mortality during the 28 y follow-up period compared to participants who never or rarely consumed olive oil [25]. Further, they reported that replacing 10 g/d of margarine, butter, mayonnaise, or dairy fat with 10 g/d of olive oil reduced cancer mortality by up to 14% [25]. Further, a meta-analysis assessing olive oil intake in 19 studies with 13,800 cancer patients and 23,340 controls reported a logOR of 0.36 for digestive cancers and 0.45 for breast cancer in participants with the highest percentile of olive oil consumption compared to the lowest [93].

Analysis of dietary intake among 450,111 participants in the EPIC cohort study found that each 10% increase in consumption of unprocessed food was associated with a 7% lower risk for CRC [35]. The World Cancer Research Fund International Continuous Update Project performed a meta-analysis exploring how specific foods and quantities affect CRC risk [36]. Their analyses found that every 90 g/day increase in whole grains reduced CRC risk by 17%, every 400 g/day increase in dairy products lowered risk by 13%, and every 100 g/day increase in fish intake lowered risk by 11% [36]. Moreover, in a study of 126 patients with CRC, survival analysis showed that a low DII score is an independent prognostic factor for overall survival (HR = 0.118) [94]. Further, an analysis of 463 participants in the Women’s Health Initiative who were diagnosed with CRC substantiated this; participants in the lowest tertile of E-DII score had a 51% lower risk for all-cause mortality during the 11.6 y follow-up period compared to those in the highest DII score tertile [95].

A prospective analysis of 110,799 UK Biobank participants utilized the Mediterranean Lifestyle index, which captures data on Mediterranean food consumption and diet habits, physical activity, rest, and social interaction habits [37]. They reported that, during a 9.4 y median follow-up period, those who had Mediterranean Lifestyle index scores in the second, third, and fourth quartiles had 10%, 17%, and 28% lower cancer mortality risk, respectively, when compared to individuals in the first quartile [37]. A study of 98,786 postmenopausal women from the Women’s Health Initiative-Observational Study used diabetes risk reduction diet scores to assess the link between diet and liver cancer [40]. The diabetes risk reduction diet score was based on intake of fiber, coffee, nuts, polyunsaturated fatty acids, red and processed meats, high-glycemic-index foods, sugar-sweetened beverages, and trans fats; a higher score was associated with a significantly lower risk of developing liver cancer during the 22 y median follow-up period [40]. Further, the authors report a significant inverse association between coffee intake and liver cancer risk [40].

Interestingly, a study in Poland of 417 women aged 40–79 y assessed the impact of nutrition knowledge on breast cancer risk and found that women who had a high level of nutrition knowledge had 49% lower odds of breast cancer [38]. Expectedly, women with higher nutrition knowledge also exhibited higher adherence to the prudent dietary pattern, which is characterized by vegetables, fruits, legumes, nuts and seeds, fish, and olive oil, and a higher MDS [38]. A meta-analysis assessing lifestyle and environmental risk factors for lung cancer reported that the Mediterranean diet, the prudent dietary pattern, the DASH diet, and carotenoid intake were associated with 13%, 20%, 13%, and 21% lower lung cancer risk, respectively [41]. Likewise, high adherence to the Mediterranean diet was associated with a 44% lower risk of head and neck cancer, according to a meta-analysis that included 11 case–control studies and 15,272 total participants [39]. Additionally, subgroup analysis revealed that high fruit intake reduced head and neck cancer risk by 33% and high vegetable intake reduced risk by 53% [39]. Interestingly, another meta-analysis of over 200,000 cancer survivors found that intake of vegetables and fish and adherence to a high-quality diet were inversely associated with overall mortality across several types of cancer [42]. In addition to reducing cancer risk and cancer mortality, high diet quality may reduce the risk and severity of treatment-induced side effects and improve response to traditional treatments. Among 132 patients with CRC in the Focus on Reducing Dose-Limiting Toxicities in Colon Cancer with Resistance Exercise trial who were receiving oxaliplatin chemotherapy, higher diet quality was associated with a significantly lower risk of moderate-to-severe chemotherapy-induced peripheral neuropathy [96]. Specifically, vegetable consumption was associated with a 71% lower risk of severe neuropathy [96]. In an analysis of NHANES data assessing cancer-related fatigue in 6413 total participants and 707 cancer patients, there was a significant inverse relationship between the Alternative Mediterranean Diet Adherence score and fatigue in both healthy participants and those with cancer [97]. Interestingly, when Mediterranean diet adherence was assessed in quartiles, fatigue was significantly lower in each incremental quartile of adherence, indicating that higher adherence to a Mediterranean diet pattern was protective against cancer-related fatigue [97].

### 3.2. Adverse Dietary Patterns

An analysis of 450,111 participants from the EPIC cohort study assessed dietary intake according to the NOVA classification and reported that a 10% higher intake of UPFs was associated with a 6% higher risk of CRC, and similarly, a 10% higher intake of processed foods was associated with a 10% higher risk of CRC [35]. Interestingly, substitution analysis revealed that substituting 10% of UPFs or processed foods with 10% unprocessed foods was associated with 6% and 10% lower CRC risk, respectively [35]. Using NOVA classification to assess UPF intake in 4870 adults, data from the MICOL and NUTRIHEP cohorts revealed that the second quartile of UPF consumption had a 65% higher gastrointestinal cancer mortality risk and the fourth quartile had a 214% higher gastrointestinal cancer mortality risk compared to the first quartile [43]. Additionally, the third quartile of UPF intake had a 61% higher risk of mortality from other cancers [43]. Another study utilizing UK Biobank data from 114,443 participants assessed whether exposure to a diet pattern associated with T2D, CVD, and all-cause mortality would also be associated with CRC [98]. The diet pattern was defined by high intake of chocolate and confectionery sugars, butter, low-fiber bread, red and processed meats, and alcohol, as well as low intake of fruits, vegetables, and high-fiber cereals; participants were divided into quintiles based on this diet pattern [98]. Participants in quintile 5 had a 34% higher risk of overall CRC and a 58% higher risk of rectal cancer when compared to participants in quintile 1 [98]. In an analysis of 126 patients with CRC, a high DII score was associated with a significantly higher postoperative complication risk and the 5-year overall survival rate was significantly lower in those in the high-DII group [94].

Diets that are high in red and processed meats and low in fibers have been shown to contribute to gut dysbiosis and increase CRC risk [99]. A meta-analysis that included 29,842 patients with CRC and 39,365 controls found significant associations between red and processed meat intake and CRC risk (OR = 1.30 and 1.40, respectively) [49]. Interestingly, this study also identified two single-nucleotide polymorphisms associated with greater CRC risk related to meat consumption [49]. A meta-analysis conducted as part of the World Cancer Research Fund/American Institute for Cancer Research Continuous Update Project (WCRF/AICR—CUP) assessed specific foods and related dose-responses and reported a 12% higher CRC risk for each 100 g/day intake of processed and red meat [36]. Likewise, every 10 g/day ethanol consumption in alcoholic beverages raised CRC risk by 7% [36]. Another WCRF/AICR—CUP study utilizing sex-specific population attributable fractions to assess cancer cases due to diet reported that a poor diet accounted for 6.3% of all cancer cases in males and 4.5% of all cases in females [50]. Particularly, processed meat consumption accounted for 10.5% of CRC cases in males and 7.0% CRC cases in females. High consumption of red meat accounted for 3.3% and 2.0% of CRC cases and low fiber intake accounted for 7.9% and 9.0% of CRC cases in males and females, respectively [50]. Additionally, a meta-analysis of 117 studies of cancer survivors reported that following a Western dietary pattern was significantly associated with a higher mortality risk across several types of cancer [42]. In addition to increasing cancer risk, poor diet quality may exacerbate the side effects of traditional cancer treatments. In patients with CRC receiving oxaliplatin chemotherapy and enrolled in the Focus on Reducing Dose-Limiting Toxicities in Colon Cancer with Resistance Exercise trial, red and processed meat consumption was associated with a 78% higher risk of moderate-to-severe neuropathy, and consumption of sugar-sweetened beverages was associated with a 57% higher risk of severe neuropathy [96].

The Women’s Health Initiative-Observational Study utilized diabetes risk reduction diet scores in 98,786 women to explore associations between diet and liver cancer risk and identified positive associations between high-glycemic-index foods and sugar-sweetened beverages and liver cancer risk [40]. The Southern Community Cohort Study, a prospective cohort study with 73,119 participants, corroborated the association between UPF consumption and liver cancer. During the 13.9 y follow-up period, participants in the highest tertile of UPF consumption had a 69% greater risk of liver cancer compared to those in the lowest tertile [44]. Specific foods that were associated with greater risk included ultra-processed grains and potatoes (29% higher risk), processed proteins (49% higher risk), and mixed dishes such as pre-prepared or frozen meals (39% higher risk). No association was observed between ultra-processed beverages and liver cancer [44].

A prospective cohort analysis of 197,426 UK Biobank participants aged 40–69 y expressed UPF consumption as a percentage of total food intake; every 10% increment in UPF consumption was associated with a 2% higher risk for overall cancer and 19% higher risk for ovarian cancer [45]. Additionally, every 10% increment in UPF intake was associated with a 6%, 30%, and 16% higher risk for mortality from overall, ovarian, and breast cancer mortality [45]. Among 150,643 women aged 50–71 y enrolled in the National Institutes of Health-AARP Diet and Health Study who were diagnosed with a primary epithelial ovarian cancer during the 20.5 y follow-up period, there was no observable association between diet quality and ovarian cancer risk [46]. However, higher prediagnosis diet quality was associated with better outcomes [46]. Specifically, a higher prediagnosis HEI-2015 score was associated with 25% lower risk of all-cause mortality, and a higher prediagnosis Alternate Mediterranean diet score was associated with lower all-cause mortality [46]. A case–control study of 62 males with prostate cancer and 63 male hospital patients without cancer controls reported that those with high UPF consumption had 2.81 times the odds of prostate cancer than those with low UPF consumption [47]. Further, data from the Cardiovascular Health Study reported that one SD increment in UPF intake resulted in a 13% higher risk of cancer mortality during the 10 y follow-up period [48].

While smoking and tobacco use are the major risk factors for lung cancer, diet quality also appears to influence risk. A secondary analysis of data from the PLCO Cancer Screening Trial, a prospective cohort study of 101,755 adults aged 55 y and above with a median follow-up of 9.4 y, revealed that E-DII scores were significantly associated with lung cancer risk [100]. Interestingly, the positive association between E-DII score and lung cancer risk is more pronounced in participants who smoked 20 or more cigarettes per day versus those who smoked less than 20 cigarettes per day, possibly indicating that a more healthful and less inflammatory diet may provide greater benefits to heavy smokers [100]. Additionally, a meta-analysis found that the Western diet pattern is associated with a 29% higher risk of lung cancer [41].

## 4. Alzheimer’s Disease and Related Dementias

Alzheimer’s disease is the leading cause of dementia and remains a leading cause of death. Pathologically, AD is defined by the presence of amyloid beta plaques and hyperphosphorylated tau tangles in the brain [101]; however, neuroinflammation and loss of brain volume are broader indicators of neurodegeneration. While there is a genetic component in some cases of AD, lifestyle habits can influence onset and rate of cognitive decline. Recent literature has proposed that AD is “type 3 diabetes”, highlighting the critical roles of insulin resistance and impaired glucose metabolism in the pathogenesis of the disease [102].

### 4.1. Protective Dietary Patterns

There is strong observational data to support that long-term healthy diet habits prevent and delay the onset of age-related neurodegenerative diseases. In 14,145 participants from the REGARDS cohort, higher adherence to the MIND diet was associated with a lower incidence of cognitive impairment in female but not male participants [51]. Data from the Personality and Total Health Through Life Cohort study indicate that greater MIND diet scores were significantly associated with lower odds of developing cognitive impairment in 1753 adults aged 60–64 y [52]. In the Rush Memory and Aging Project, a prospective study of 923 adults aged 58–98 y living in retirement communities and senior public housing units, only the third tertile for DASH and Mediterranean diet was associated with lower AD risk after the 4.5 y follow-up, indicating that high adherence to these diets may be necessary to attain cognitive benefits [53]. However, both the second and third tertiles of adherence to the MIND diet were associated with lower AD incidence, suggesting that moderate adherence to the MIND diet may be sufficient to decrease AD risk [53]. An analysis of NHANES data from 27,773 participants found that during a median follow-up of 9.8 y, a higher Alternative Mediterranean Diet Adherence score was associated with a lower risk of AD mortality [58].

An analysis of UK Biobank data utilized quartile analysis to assess adherence to the Recommended Food Score (which evaluates intake of fruit, vegetables, whole grains, meat and alternatives, and reduced-fat dairy products), the MDS, and the MIND diet. The results revealed that among 121,521 participants, the highest quartile of adherence to any of the three dietary patterns was associated with a lower risk of all-cause dementia and AD, and high adherence to the Mediterranean Diet or MIND diet was associated with a lower risk of vascular dementia [59]. Further, in 131,209 UK Biobank participants aged 40–69 y, greater adherence to the Mediterranean diet and MIND diet resulted in 21% and 27% lower dementia risk, respectively, over the 13.5 y follow-up period [54]. Furthermore, a higher Recommended Food Score lowered dementia risk by 28% and a higher AHEI score lowered risk by 23% [54]. In an analysis of 92,849 participants aged 45–75 y from the Multiethnic Cohort Study, higher baseline scores for adherence to the Mediterranean, DASH, or MIND diets, and higher HEI-2015 scores were all associated with significantly lower risk of AD and related dementias [55]. Notably, the associations were more pronounced in African American, Latino, and White participants than in Japanese American and Native Hawaiian participants. Further, over the 10 y follow-up period, this study reported that improving diet quality was associated with lower risk of AD and related dementias in participants of all ages, indicating that adopting a high-quality diet is beneficial at any point in an individual’s life [55].

A secondary analysis of the Health and Retirement Study data revealed that adherence to the Mediterranean, DASH, or MIND diet at baseline was significantly associated with baseline cognition, and adherence to the Mediterranean or DASH diet was associated with slower cognitive decline over the 6 y follow-up period [56]. Moreover, in the Personality and Total Health Cohort Study, higher adherence to the DASH or Mediterranean diet was significantly associated with higher Mini-Mental State Examination (MMSE) scores, indicating higher cognitive function [52]. The Cache County Study on Memory, Health and Aging included 3831 adults aged ≥65 y and corroborated this finding; participants in the highest quintiles of adherence to a DASH or Mediterranean diet had significantly higher Modified Mini-Mental State Examination (3MS) scores, and this was observed consistently during an 11 y follow-up period [103]. Further, this study reported that higher consumption of whole grains, nuts, legumes, and vegetables was significantly associated with higher 3MS scores, indicating better cognitive function. Similarly, a secondary analysis of ASPREE and ALSOP data revealed significantly higher 3MS scores among participants in the highest quartile of MDS when compared to those in the lowest quartile [24]. An analysis of data from 1907 adults without dementia aged ≥60 y enrolled in the Swedish National Study on Aging and Care in Kungsholmen assessed associations between diet patterns and AD blood biomarkers [60]. Participants in the highest tertile of adherence to the Mediterranean diet had significantly lower phosphorylated (p)-tau 181 compared to participants in the lowest adherence tertile, delineating another mechanism by which diet may impact cognition [60]. In addition, there were significantly fewer participants with an MMSE score < 27 in the highest tertile of Mediterranean diet adherence when compared to the lowest [60]. Another longitudinal study of 5705 adults confirmed reduced dementia risk and improved cognitive function in participants who were more adherent to the Mediterranean diet, and interestingly, this relationship was stronger in *APOE4* homozygotes [61]. Furthermore, in *APOE4* homozygotes, the Mediterranean diet more effectively altered plasma metabolites, including cholesterol esters, sphingomyelins, and glycerides, related to dementia risk [61].

An observational study of 92,383 adults assessed the influence of olive oil consumption on cause-specific mortality and found that respondents consuming > 7 g/day of olive oil had a 29% lower risk of neurodegenerative disease mortality compared to those who never or rarely consumed olive oil over a 28 y follow-up period [25]. Additionally, substitution analysis revealed that replacing 10 g/d of margarine, butter, or mayonnaise with an equivalent amount of olive oil reduced neurodegenerative disease mortality by up to 20%. An analysis of the Framingham Offspring Cohort data from 1644 adults aged ≥60 y found that greater adherence to the MIND diet was associated with lower risk of dementia over the 14 y follow-up period, slower biological aging as measured by DunedinPACE, and slower DunedinPACE was associated with lower risk for dementia and mortality [57].

RCTs assessing the impact of nutrition interventions on cognition are limited. In a two-site RCT with 604 cognitively normal adults aged ≥65 y with a family history of dementia, there was no difference in cognition or brain imaging outcomes after 3 years of following the MIND diet versus mild calorie restriction [104]. Notably, the Finnish Geriatric Intervention Study to Prevent Cognitive Impairment and Disability (FINGER) trial was a large RCT in older adults at high risk for dementia that utilized a 2-year multidomain lifestyle intervention, including a nutrition intervention based on the Finnish Nutrition Recommendations, to prevent cognitive decline [105]. Comparably, the US Study to Protect Brain Health Through Lifestyle Intervention to Reduce Risk (POINTER) trial was another large RCT employing a 2-year multidomain lifestyle intervention, including the MIND diet, in at-risk older adults [105]. Both the FINGER and POINTER trials were successful at improving cognitive function in the intervention group; however, because they utilized multidomain interventions, it is not possible to delineate the exact impact of the nutrition component of the intervention.

### 4.2. Adverse Dietary Patterns

There is evidence indicating that pro-inflammatory dietary patterns can negatively affect cognitive function in older adults. In 7085 women aged 65–79 y enrolled in the Women’s Health Initiative Memory study, higher DII scores at baseline were associated with greater cognitive decline and earlier onset of cognitive impairment during a 9.7 y follow-up period [106]. Similarly, a cross-sectional analysis of data collected from the Microbiome in Aging Gut and Brain consortium cohort of 217 adults aged ≥60 y demonstrated that participants with cognitive impairment had elevated inflammatory markers and higher levels of intestinal inflammation, as indicated by higher stool calprotectin [107]. Further, DII scores were weakly but significantly correlated with stool calprotectin levels, identifying a possible mechanism by which an inflammatory diet may contribute to cognitive decline [107]. Furthermore, a prospective cohort study explored the impact of the oral–gut–brain axis on cognitive function in 511 adults aged ≥65 y without dementia [108]. They reported that a one-unit increase in empirical-DII score was associated with poor memory performance, and this association was stronger in those with periodontitis and *Helicobacter pylori* infection [108]. Among 131,209 UK Biobank participants aged 40–69 y, a higher energy-adjusted DII score was associated with a 30% higher dementia risk during a median follow-up on 13.5 y [54]. Another analysis of the UK Biobank data in 58,423 participants aged 40–70 y indicated that UPF intake was associated with higher risks of incident dementia, Parkinson’s Disease, and multiple sclerosis [62]. Additionally, greater UPF intake was associated with substantial gray matter loss [62]. Likewise, a cross-sectional analysis of ASPREE and ALSOP data found that older participants in the highest quartile of UPF consumption had significantly lower 3MS scores when compared to those in the lowest quartile of UPF intake [24].

## 5. Type 2 Diabetes and Insulin Resistance

Type 2 diabetes (T2D) is the seventh leading cause of death in the United States, and lifestyle habits play a major role in the development of prediabetes and T2D. Importantly, insulin resistance and T2D are major risk factors for other chronic diseases, including CVD, Alzheimer’s disease, and fatty liver disease. Notably, dietary patterns and other lifestyle behaviors can halt or reverse the progression of prediabetes and T2D.

### 5.1. Protective Dietary Patterns

The CORDIOPREV study investigated the impact of a Mediterranean diet versus a low-fat diet in 183 patients with T2D [63]. Responders (i.e., those who achieved T2D remission) exhibited lower inflammation after the 5-year intervention period, as indicated by a significant decrease in neutrophil counts, compared to non-responders (i.e., those who did not achieve remission) [63]. Further, responders had a significant improvement in insulin sensitivity and β-cell function after the 5-year intervention period [63]. Interestingly, lower neutrophil counts at baseline were significantly associated with a higher likelihood of achieving T2D remission when compared with higher neutrophil counts at baseline [63]. Additionally, secondary analysis of ASPREE and ALSOP data found that fasting glucose and the prevalence of T2D were lower among participants in the highest quartile of MDS compared to those in the lowest quartile [24]. A randomized, crossover feeding trial in 89 participants (mean age 67 y) with T2DM provided participants with four isocaloric diets for 5 weeks each: the DASH4D diet (a DASH-like diet specifically designed for diabetes) with high sodium (3700 mg/day), the DASH4D diet with low sodium (1500 mg/day), a high-sodium (3700 mg/day) comparison diet, or a low-sodium (1500 mg/day) comparison diet. Expectedly, the DASH4D diet resulted in a significant decrease in mean glucose levels (averaging between 70 and 180 mg/dL) and an increase in time-in-range glucose levels, regardless of sodium content [64]. An analysis of 6798 participants in the Rotterdam Study, a prospective cohort study in the Netherlands, investigated the relationships between a plant-based versus an animal-based diet and insulin resistance, prediabetes, and T2D. Interestingly, after accounting for sociodemographic and lifestyle factors, a higher plant-based dietary index score was associated with lower insulin resistance based on homeostatic model assessment of insulin resistance (HOMA-IR), an 11% lower risk of prediabetes, and an 18% lower risk of T2D [65].

### 5.2. Adverse Dietary Patterns

UPF consumption and diet-induced inflammation may increase T2D risk. Data from the Nurses’ Health Study, the Nurses’ Health Study II, and the Health-Professionals Follow-up Study were analyzed to explore the impact of UPF consumption on T2D risk; in 198,636 participants across all cohorts, the highest quintile of UPF intake had a 28% greater risk of T2D when compared to the lowest [66]. Specifically, ultra-processed refined breads, sauces, spreads, and condiments, packaged savory snacks, artificially and sugar-sweetened beverages, animal-based products, ready-to-eat dishes, and other ultra-processed foods were associated with significantly higher T2D risk [66]. The ELSA-Brasil study found that one SD higher UPF consumption was associated with an 11% higher risk for diabetes, as indicated by a relative risk of 1.11 [30]. Moreover, consumption of processed meats and sweetened beverages was associated with a 7% and 14% significantly greater risk of diabetes, respectively [30]. Further, analysis of UK Biobank data from 90,631 participants found that participants with high UPF consumption had a 36% higher risk of type 2 diabetes compared with those with low UPF intake [31]. Each 10% increase in UPF intake was associated with a 10% higher T2D risk, indicating a dose-dependent relationship [31]. Further, they found that the risk of T2D was 301% greater, as indicated by a HR of 4.01, in participants who had high UPF consumption and higher genetic predisposition, based on polygenic risk score [31].

An analysis of NHANES data on 16,512 adults aged ≥60 y found that participants in the highest DII quartile had 17% higher odds of having diabetes than those in the lowest quartile [90]. A large comparative study of 69,728 adults aged 18–69 y utilized four validated T2D risk scores (QDscore, Finrisk, Canrisk, and TRAQ-D) to assess the impact of sociodemographic and lifestyle factors on T2D risk in the industry and commerce sectors [109]. Among all participants, poor adherence to the Mediterranean diet was associated with significantly greater odds of T2D across all T2D risk scores (OR = 2.82–6.62) [109]. A meta-analysis of 31 cohort studies that included 1,966,444 participants identified significant associations between meat consumption and T2D risk [67]. Specifically, consuming 100 g/day of unprocessed red meat was associated with 10% higher risk of T2D, 50 g/day processed meat was associated with 15% higher risk, and 100 g/day poultry was associated with 8% higher risk [67]. Furthermore, replacing processed meat with unprocessed red meat or poultry was associated with 7 or 10% lower T2D risk, respectively [67].

## 6. Frailty

Frailty is common in older adults and can lead to decreased independence and increased vulnerability to adverse health complications. Frailty is characterized by declining physiological function and can be defined by weakness, slow walking speed, exhaustion, low physical activity, and unintentional weight loss. Sarcopenia is the age-related progressive loss of muscle mass, strength, and function and often occurs with or contributes to the development of frailty. Lifestyle plays a substantial role in the development of frailty; chronic inflammation contributes to frailty and sarcopenia and represents a mechanism by which diet may exacerbate or reduce risk.

### 6.1. Protective Dietary Patterns

Consumption of unprocessed and minimally processed foods may be protective for nutritional frailty. In the Salus in Apulia Study in Italy, adults aged ≥65 y in the highest quintile of unprocessed and minimally processed food consumption had 90% lower odds for nutritional frailty [68]. An analysis of 7300 NHANES participants aged ≥60 y found 29% or 48% significantly lower frailty odds in participants who had either moderate or high adherence to an alternate Mediterranean diet, respectively [69]. Hip fractures are a major health risk in frail older adults. In the Consortium on Health and Aging: Network of Cohorts in Europe and the United States (CHANCES) project, a cohort study of 140,775 older adults, a 2-point higher MDS was associated with a significant 4% lower hip fracture risk, signifying that enhanced adherence may elicit greater benefit [70]. A secondary analysis of ASPREE and ALSOP data revealed that high adherence to the Mediterranean diet reduced frailty risk in older adults; specifically, participants in the highest quartile of MDS had significantly less frailty and prefrailty than those in the lowest quartile [24]. Moreover, when assessing MDS as a continuous variable, those with higher adherence had a 12% lower risk of frailty and a 7% lower risk of prefrailty [24]. In the Women’s Health Initiative Long Life Study, a longitudinal study of 4516 community-dwelling women aged ≥65 y, a higher MDS at baseline was associated with better physical function; however, higher diet adherence did not significantly impact the rate of physical function decline over the 8 y follow-up period [71].

### 6.2. Adverse Dietary Patterns

An analysis of 15,249 NHANES participants aged ≥20 y found that higher E-DII scores and lower HEI-2015, MDS, and DASH scores were significantly associated with higher frailty [110]. A meta-analysis evaluating the association between DII and frailty in older adults recently reported that individuals in the highest DII category had a 47% higher risk of frailty and a 54% higher risk of pre-frailty compared to those in the lowest DII category [111]. Likewise, 2795 adults aged ≥60 y with T2D from the NHANES cohort in the second and third tertile of DII scores had 36% and 33% greater odds of frailty, respectively, compared to the first tertile [112]. A case–control study of 160 older adults in Iran, with (case) or without (control) sarcopenia, showed that each increment in DII score was associated with 12.9% higher odds of sarcopenia, corroborating that an inflammatory diet likely contributes to sarcopenia and frailty [113]. A study of new-onset frailty in 811 community-dwelling older adults enrolled in the Kashiwa cohort study supported this; participants in the highest tertile of DII scores had more than a 2-fold higher risk of developing frailty compared to those in the lowest DII tertile [114]. Further, an analysis of 1586 older adults in NHANES reported a significant association between E-DII scores and frailty status in older adults with fatty liver disease [115]. A cross-sectional analysis of 249 patients with T2D (mean age 62.1 y) found that DII scores were significantly higher in adults with sarcopenia compared to those without sarcopenia, and that a higher DII score is an independent risk factor for sarcopenia [116]. Further, handgrip strength and appendicular muscle mass were significantly lower in each tertile of higher DII scores [116].

A cross-sectional analysis of nutritional frailty and processed food intake in older adults in the Salus in Apulia Study reported odds ratios of 1.46 and 3.22 in the fourth and fifth highest quintiles of processed food consumption, respectively, when compared to the first quintile [68]. Similarly, an analysis of ASPREE and ALSOP data assessing UPF consumption as a continuous variable reported an 11% greater risk for frailty and a 5% greater risk for prefrailty in participants with higher UPF intake [24]. Among 1973 Spanish adults aged ≥60 y enrolled in the Seniors-ENRICA cohort study, those in the highest tertile of added sugar consumption had a 93% greater risk of unintentional weight loss and 2.27 times higher odds of developing frailty compared to those in the lowest tertile [73]. Another analysis of 1822 older adults in the Seniors-ENRICA cohort study corroborated that UPF consumption can contribute to greater frailty risk; when assessed as a percentage of total energy intake, participants in the second, third, and fourth quartiles of UPF intake had 1.52, 2.98, and 3.67 higher odds of frailty when compared to those in the first quartile [72].

## 7. Liver Disease

Liver disease continues to be a global health burden. Non-alcoholic fatty liver disease (NAFLD), recently renamed metabolic dysfunction-associated steatotic liver disease (MASLD), is the most common form of liver disease and affects over 30% of the population worldwide. Aging is a significant risk factor for liver disease [117]. Dietary factors play a critical role in the development and progression of liver diseases, with certain dietary habits increasing risk and others providing protective benefits.

### 7.1. Protective Dietary Patterns

An analysis of 98,786 postmenopausal women in the Women’s Health Initiative found that adherence to a diabetes risk reduction diet was associated with significantly reduced risks of chronic liver disease mortality and liver cancer incidence during a median 22 y follow-up period [40]. Participants in the highest tertile of diet adherence had a 46% lower risk of chronic liver disease mortality compared to those in the lowest tertile of diet adherence [40]. Interestingly, participants in the middle tertile were also found to have lower risk compared to the bottom tertile, proposing that even moderate adherence to a diabetes risk reduction diet may help to lower the risk of chronic liver disease mortality [40]. Notably, the dietary component most strongly associated with chronic liver disease mortality was high fiber intake; participants in the top tertile of fiber intake had a 51% lower risk [40].

In a post hoc analysis of data from 16,703 participants from the ASPREE study, greater adherence to a Mediterranean diet was associated with significantly lower risk of MASLD in adults aged ≥70 y [74]. Participants in the highest tertile of Mediterranean diet adherence were found to have 10% lower prevalence of MASLD compared to the lowest tertile [74]. Importantly, the protective nature of the Mediterranean diet was still present even in participants who had high UPF intake [74], highlighting that overall dietary quality is very important. Furthermore, the benefits of the Mediterranean diet were highlighted in a recent analysis of data from 84,024 participants in the Million Veteran Program; specifically, adherence to a diet higher in nutrients and foods classically associated with the Mediterranean diet was associated with a significantly less fibrosis progression in participants with MASLD [75]. Additionally, an RCT exploring the effects of olive oil consumption in 66 patients with NAFLD demonstrated that daily consumption of extra-virgin olive oil (20 g/day) for 12 weeks significantly reduced fatty liver grade when compared to consumption of sunflower oil [76]. Analysis of UK Biobank data from over 487,000 individuals found that oily fish intake was protective against severe metabolic-associated fatty liver disease (MAFLD) and lowered risk by 28% over the 12.1 y follow-up period [77].

### 7.2. Adverse Dietary Patterns

Mounting evidence indicates that diets high in UPFs are associated with elevated liver disease risk. One study utilizing UK Biobank data from 143,073 participants aged 40–69 y expressed UPF consumption as a percentage of total energy intake; every 10% increment in UPF intake was associated with a 9% higher risk of severe NAFLD [78]. Moreover, participants in the highest quartile of UPF intake had a 26% greater risk of developing severe NAFLD during the 10.5 y follow-up period compared to those in the lowest quartile of UPF intake [78]. Subgroup analysis revealed that participants with a BMI ≥25 kg/m^2^ were at a heightened risk of severe NAFLD due to high UPF intake [78]. A meta-analysis of 9 studies that included 60,961 participants aged ≥18 y found that high intake of UPFs resulted in 42% higher NAFLD risk, while moderate intake resulted in a 3% higher risk [79]. An analysis of older adults from the ASPREE cohort found that higher UPF intake was significantly associated with greater MASLD risk; participants in the highest and second tertiles of UPF consumption had a 14% and 13% higher MASLD risk compared to those in the lowest tertile, respectively [74]. Similarly, an analysis of 6545 NHANES participants found that patients with NAFLD consume more UPFs than controls, and expectedly, UPF consumption was inversely associated with HEI-2015 score [80]. Further, individuals in the highest quartile of UPF consumption had 83% higher odds of having NAFLD compared to those in the lowest quartile, and every 10% increment in UPF intake resulted in 15% higher odds of NAFLD [80].

Interestingly, a cross-sectional analysis of 44,624 Korean adults aged 40–69 y in the Health Examinees Study highlighted sex-specific differences in the relationship between UPF intake and NAFLD risk [81]. Women in the highest quartile of UPF consumption had a 48% greater risk of NAFLD, whereas men in the highest quartile had a 35% greater risk [81]. An analysis of 487,875 participants in the UK Biobank reported that during the 12.1 y follow-up period, consuming meat ≥7 times per week was associated with a 76% higher risk of MAFLD, and consuming processed meat >3 times per week was associated with a 19% higher risk [77]. According to the Framingham Heart Study, which analyzed data from the Framingham Offspring cohort (mean age 63 y) and the Framingham Third Generation cohort (mean age 48 y), the authors reported that participants in the older Offspring cohort who consumed sugar-sweetened beverages were at greater risk for developing NAFLD, while the younger Third Generation cohort was not affected [82]. Additionally, older Offspring cohort participants who consumed sugar-sweetened beverages had more liver fat compared to non-consumers [82]. Likewise, the ELSA-Brasil longitudinal study found that one SD increment in UPF intake results in a 12% higher MASLD risk, and consumption of processed meats, sweetened beverages, and baked and fried snacks was associated with a significantly greater risk of MASLD during the ~8 y follow-up period [30].

## 8. Conclusions and Future Directions

The evidence reviewed here indicates that adherence to a healthful diet pattern has the potential to reduce chronic disease risk and mortality significantly. Interestingly, data on several different dietary patterns and various chronic diseases show that individuals at the highest risk, such as heavy smokers, those with a high BMI, or *APOE4* homozygotes, may derive the greatest benefits from favorable dietary patterns and dietary interventions. These findings highlight the potential impact and importance of a healthful diet.

A strength of the present review is that many of the studies have large sample sizes and focus on hard outcomes; however, these studies do not provide causal evidence between nutrition patterns and health outcomes. Notably, a major challenge in assessing diet quality is potential inaccuracy of self-reported food consumption. Additional considerations for future work include the influence of nutraceuticals (e.g., soy isoflavones, curcumin, probiotics), plant sterols and stanols, and dietary supplements. Further, social determinants of health are an important consideration when discussing diet quality, as income, education, occupation, and geographic location influence ability to adopt or maintain a healthful dietary pattern. Lack of nutrition education is another major barrier to consider.

The mechanisms by which nutrition patterns influence health are not entirely understood. Future research is important to continue to explore the controversial influence of specific dietary components and the impact of various food processing techniques on diet quality. Additionally, identifying strategies to optimize adoption of and adherence to a healthful dietary pattern is essential. Likewise, efforts to mitigate potentially harmful dietary choices are equally important. Importantly, future studies should focus on the degree of adherence necessary to elicit the desired benefits. Further, a dose-dependent relationship between dietary adherence and benefits seems apparent; thus, identifying strategies to help participants follow a healthful diet is important to fully understand the potential impact.

## Figures and Tables

**Table 1:** Protective and adverse nutrition patterns and foods that prevent or contribute to noncommunicable disease risk. Various diet patterns and specific foods may increase or decrease the risk of several age-related chronic diseases. The bracket numbers represent references.

Noncommunicable Disease/Condition	Protective Dietary Patterns and Foods	Adverse Dietary Patterns and Foods
Cardiovascular disease	Mediterranean diet [22,23,24] >7 g olive oil/day [25] Mediterranean diet supplemented with nuts or olive oil [26] Vegetable protein [27] Fiber intake [27] Unprocessed poultry >4×/week [28] Dairy products [29]	Ultra processed food [24,30,31,32,33,34] Processed meat >4×/week [28] Animal proteins [27] Distilled alcoholic beverages [30] Sweetened beverages [30]
Cancer	>7 g olive oil/day [25] Unprocessed foods [35] 90 g/day whole grains [36] 400 g/day dairy products [36] 100 g/day fish [36] Mediterranean diet [37,38,39] Diabetes risk reduction diet [40] Coffee [40] Prudent dietary pattern [38] DASH diet [41] Carotenoid intake [41] Fruit and vegetable intake [42]	Ultra processed food [35,43,44,45,46,47,48] Red meat [36,49] 100 g/day processed meat [36,49] 10 g/day ethanol (alcohol) consumption [36] Low fiber [50] Western diet [41] Sugar-sweetened beverages [40] High-glycemic foods [40]
Alzheimer’s disease and related dementias	MIND diet [51,52,53,54,55,56,57] DASH diet [52,53,55,56] Mediterranean diet [24,53,54,55,56,58,59,60,61] >7 g olive oil/day [25]	Ultra processed food [24,62]
Type 2 diabetes	Mediterranean diet [24,63] DASH4D diet [64] Plant-based foods [65]	Ultra processed food [30,31,66] 50 g/day processed meats [30,67] Sugar-sweetened beverages [30,66] 100 g/day red meat [67]
Frailty	Unprocessed and minimally processed food [68] Mediterranean diet [24,69,70,71]	Ultra processed food [24,68,72] Added sugar [73]
Liver disease	Diabetes risk reduction diet [40] Fiber intake [40] Mediterranean diet [74,75] 20 g/day olive oil [76] Oily fish [77]	Ultra processed food [30,74,78,79,80,81] Meat ≥7×/week [77] Processed meat >3×/week [77] Sugar-sweetened beverages [82]

## Data Availability

Data sharing is not applicable.
